# Hospitalization Records as a Tool for Evaluating Performance of Food- and Water-Borne Disease Surveillance Systems: A Massachusetts Case Study

**DOI:** 10.1371/journal.pone.0093744

**Published:** 2014-04-16

**Authors:** Siobhan M. Mor, Alfred DeMaria Jr., Elena N. Naumova

**Affiliations:** 1 Farm Animal and Veterinary Public Health, Faculty of Veterinary Science, The University of Sydney, Sydney, New South Wales, Australia; 2 Marie Bashir Institute for Infectious Diseases and Biosecurity/School of Public Health, The University of Sydney, Sydney, New South Wales, Australia; 3 Bureau of Infectious Diseases, Department of Public Health and Community Medicine, School of Medicine, Tufts University, Boston, Massachusetts, United States of America; 4 Massachusetts Department of Public Health, Boston, Massachusetts, United States of America; 5 Department of Civil and Environmental Engineering, School of Engineering, Tufts University, Medford, Massachusetts, United States of America; California Department of Public Health, United States of America

## Abstract

We outline a framework for evaluating food- and water-borne surveillance systems using hospitalization records, and demonstrate the approach using data on salmonellosis, campylobacteriosis and giardiasis in persons aged ≥65 years in Massachusetts. For each infection, and for each reporting jurisdiction, we generated smoothed standardized morbidity ratios (SMR) and surveillance to hospitalization ratios (SHR) by comparing observed surveillance counts with expected values or the number of hospitalized cases, respectively. We examined the spatial distribution of SHR and related this to the mean for the entire state. Through this approach municipalities that deviated from the typical experience were identified and suspected of under-reporting. Regression analysis revealed that SHR was a significant predictor of SMR, after adjusting for population age-structure. This confirms that the spatial “signal” depicted by surveillance is in part influenced by inconsistent testing and reporting practices since municipalities that reported fewer cases relative to the number of hospitalizations had a lower relative risk (as estimated by SMR). Periodic assessment of SHR has potential in assessing the performance of surveillance systems.

## Introduction

Foodborne illness affects an estimated 47.8 million people in the United States each year, causing more than 128,000 hospitalizations and 3,000 deaths [1], [2]. Similarly, waterborne illness is estimated to affect 19.5 million residents each year [3]. The total health-related cost of foodborne illness is likely to be as much as $77 billion per year [4], while hospitalization costs associated with three common waterborne pathogens alone cost the healthcare system an estimated $539 million annually [5].

In the United States, surveillance for notifiable diseases – including those transmitted via food and water – falls within the mandate of local and state governments. These surveillance systems typically rely on passive reporting by laboratories and healthcare providers who notify local health authorities when cases are diagnosed. In Massachusetts, diseases are reportable to the local board of health (BOH), of which there are 351. BOHs receive clinician or laboratory reports through a variety of means, and are responsible for completing investigations to classify cases as confirmed, probable, or not a case. To improve performance of surveillance systems, automated electronic laboratory reporting methods are increasingly being used in Massachusetts and elsewhere [6]–[8]. Furthermore, national surveillance systems are expanding the number and range of proxies evaluated to better characterize the burden of infection and performance of public health surveillance [9], [10].

Cases detected through passive surveillance represent only a fraction of the “true” number of cases that occur in the population because: (1) most patients have mild symptoms of short duration and so do not seek medical care; (2) of those that do access clinical care, many will not have the laboratory test performed to determine a specific etiology; and (3) of those who seek medical care in whom an etiologic diagnosis is confirmed, not all will be reported to the surveillance system(s) [11]. Importantly, points (2) and (3) represent potential areas where targeted interventions by public health authorities (e.g. training BOH members and physicians) might be reasonably expected to yield improvements in surveillance, provided jurisdictions under-ascertaining cases can be identified. This remains challenging in the absence of data on the “true” number of cases in each reporting jurisdiction.

In this paper we outline a framework for evaluating jurisdiction performance by comparing surveillance and hospitalization records for the same illness. While hospitalizations represent a fraction of the total number of cases of illness in the population, gastrointestinal illness severe enough to require hospitalization should result in a diagnostic evaluation likely to identify an etiologic agent should it be present. Using Massachusetts as a case study, we show that comparison of surveillance and hospitalization data could be a useful tool for identifying geographic areas that deviate from the typical experience and might be under-ascertaining cases of reportable disease.

## Materials and Methods

### Theoretical framework

To formulate the analytical plan we designed a theoretical framework that considers the natural history of enteric infections, surveillance system attributes, and health care utilization. The spectrum of gastrointestinal disease is frequently described as an “iceberg” or “pyramid”. The shape of the pyramid – that is the relative number of cases that are mild, moderate and severe, for any particular pathogen – is not usually known. Typically, asymptomatic and mild cases of disease are difficult to enumerate without laboratory studies to confirm an etiologic diagnosis. Instead, most surveillance systems rely on detection of cases that seek medical assistance and are subsequently diagnosed with infection because of a positive laboratory test, a subset of which will be hospitalized due to severe illness. For a pathogen of moderate virulence, a relatively small proportion of those that seek medical attention will be hospitalized. Thus, the ratio between the number of cases reported through surveillance and the number of hospitalizations observed over the same time period for the same infection (surveillance to hospitalization ratio, SHR) should be well in excess of 1. In the ideal scenario, the magnitude of the ratio represents biological processes only. For instance, infection with high virulence organisms or pathogens that are of significant interest, such as *Salmonella* Typhi, might be expected to result in hospitalization in many cases, especially in the elderly, and reporting of most cases identified. In this situation the SHR will approach 1. Alternatively, variations in the ratio might reflect artifacts of the surveillance system. For example, incomplete reporting will artificially decrease this ratio if not all identified cases are reported; in the worst case scenario, this ratio may fall below 1 if not all hospitalized cases are reported.

### Data

We used temporally aggregated data from Massachusetts to examine the utility of hospitalization records as a tool for evaluating food- and waterborne surveillance systems. While the analysis focused on surveillance and hospitalization for salmonellosis, we abstracted data on other food- and waterborne infections to explore potential differences between pathogens. A limited dataset comprising surveillance records for the period January 1991 to December 2004 (January 1995–December 2004 for *Cryptosporidium*) was obtained from the Massachusetts Department of Public Health (MDPH), Division of Epidemiology and Immunization. Criteria for reporting to MDPH during that time period included: isolation of a specific microorganism from any clinical specimen (*Campylobacter*, *Salmonella* and *Shigella*); demonstration of immunoglobulin M antibody to hepatitis A virus in the blood; or demonstration of parasites or antigen in stool, intestinal fluid or small-bowel biopsy specimens (*Cryptosporidium* and *Giardia*) [12]. All records for Massachusetts residents aged ≥65 years were extracted from the database, including municipality of residence.

Hospitalization discharge data was obtained from the Centers for Medicare and Medicaid Services (CMS) for the corresponding 14 year period. The Medicare Provider Analysis and Review database contained information on age, sex, date of hospitalization or emergency room visitation, zip code of residence and up to 10 diagnostic codes (classified by the International Classification of Disease, 9th edition, with Clinical Modification [ICD-9-CM]). We extracted all records for Massachusetts residents aged ≥65 years containing codes for notifiable food- and water-borne diseases, namely infection with *Campylobacter* (ICD-9-CM 008.43), *Cryptosporidium* (007.2, 007.4, 007.8, 007.9), *Giardia* (007.1), hepatitis A (007.0, 007.1), non-typhoid *Salmonella* (003.X), *Salmonella* Typhi (002.0), and/or *Shigella* (004.X). An expanded case definition was used for cryptosporidiosis based on prior evidence of misclassification of this infection [13].

### Analysis

Annual incidence of infection was calculated for each notifiable disease and for each data source (surveillance and hospitalization) using elderly population data derived from the 1990 and 2000 decennial census. The elderly population was estimated by linear interpolation between census years, with the reported annual incidence values based on the elderly population at the midpoint of the study period (1997/1998; 1999/2000 for *Cryptosporidium* surveillance). The crude SHR was calculated for each notifiable disease by dividing the observed number of cases reported through surveillance (So) by the observed number of hospitalized (Ho) cases.

For diseases with sufficient counts – namely, salmonellosis, campylobacteriosis and giardiasis – the observed number of cases reported through surveillance (So) and the observed number of hospitalized cases (Ho) were aggregated by municipality. To explore spatial differences in relative risk as depicted by the surveillance system, we calculated the crude standardized morbidity ratio (SMR) for each municipality by dividing the observed (So) and the expected (Se) number of reported cases (So/Se) [14]. Expected values (Se) were calculated by multiplying the elderly population (aged ≥65 years) in each municipality by the mean incidence for the entire state of Massachusetts. The latter was calculated by dividing the total number of cases reported through surveillance by the total population aged ≥65 years in Massachusetts.

Markov Chain Monte Carlo (MCMC) methods were employed to construct Bayesian credible intervals to reflect the degree of uncertainty for point estimates of SMR. Smooth point estimates and uncertainty measures were compiled by a large number of simulations implemented in the MCMC modeling algorithm, applying a standard Poisson-gamma model as follows [15], [16]:
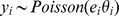


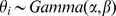
where *y_i_* is the observed number of cases reported through surveillance (So) in town *i*, *e_i_* is the expected number of cases reported through surveillance (Se), and *θ_i_* is the ratio between *y_i_* and *e_i_* in town *i*. Thus, *θ_i_* is an estimate of the mean SMR in town *i*. Model convergence was assessed through examination of trace plots and Gelmen-Rubin statistics. Posterior estimates of the mean and associated 95% Bayesian credible interval were generated for the entire state and for each town. The latter were mapped using ArcGIS software (version 9.3.1; Esri, Redlands CA). By convention, a town was deemed to have a higher or lower risk than expected if the 95% credible interval for the SMR excluded 1 (obtained when observed and expected values are the same).

Similarly, the ratio between the observed number of reported and hospitalized cases (So/Ho) was calculated from the aggregated data, providing a crude estimate of the SHR in each municipality. To generate uncertainty measures for SHR, we repeated the above MCMC procedures using the observed number of hospitalized cases (Ho) in place of the expected value, *e_i_*. Thus, in this context, *θ_i_* is an estimate of the mean SHR in town *i*. For this model, analysis was limited to only those municipalities with ≥1 hospitalized case. Posterior estimates of the mean SHR and uncertainly measures were generated and mapped as described above. A municipality was deemed to be suspect of under-reporting if the point estimate for SHR was below the state average and the 95% credible interval for the municipality did not overlap with the 95% credible interval for the entire state. The number and per cent of municipalities with suspected under-reporting was estimated for each infection.

Finally, correlation and multiple regression analyses were conducted to assess spatial similarity and the extent to which spatial heterogeneities in SMR can be predicted by SHR, adjusting for differences in elderly population age-structure. This approach assumes that: a) for a given infection, spatial heterogeneity in relative risk detected through surveillance includes both “true” variation in incidence and artifacts related to differences in testing procedures and reporting and is depicted by SMR = So/Se; and b) for a given infection and within an age-standardized population, the relationship between the number of cases reported through surveillance and those that are hospitalized reflects spatial heterogeneity in testing procedures and mandatory reporting (contributing to variable data quality) and is depicted by SHR = So/Ho. Similarity between spatial distributions of these two measures could therefore be indicative of a unique latent spatial process. Positive associations depicted by correlation coefficients between SMR and SHR indicate whether lower relative risk coincide with underreporting in reference to mandatory requirements. For all regression models, smoothed estimates of SMR and SHR for each jurisdiction were log-transformed and used as the dependent and independent variables, respectively. Models were adjusted for the proportion of elderly persons aged 75–84 years and 85 years and above in each jurisdiction (at the 2000 decennial census). Observations with large residuals were examined and excluded from subsequent analyses if they were deemed potentially influential. Quality of fit was reported as adjusted R^2^.

Data analysis was performed in R (version 2.13.0; R Foundation for Statistical Computing, Vienna Austria) with MCMC implementation performed in WinBUGS [17] (version 1.4.3) using the R2WinBUGS package [18]. This study was reviewed and exempted by the Institutional Review Board at Tufts Medical Center and Tufts University Health Sciences Campus. Use of the surveillance and hospitalization data was permitted through data sharing agreements between Tufts and MDPH and CMS, respectively.

## Results

Notifiable disease counts and annual rate of disease by etiology and data source are shown in Table 1 along with the crude SHR for each infection. *Campylobacter* and non-typhoid *Salmonella* were the most common etiologies, while typhoid was (expectedly) the least common.

**Table 1:** Notifiable food- and waterborne diseases in Massachusetts, 1991–2004*.

Disease	Surveillance		Hospitalization		SHR
	No. of cases	Annual incidence†	No. of cases	Annual incidence†	
Cryptosporidiosis*	30	0.4	57	0.5	0.5
Typhoid	7	0.1	7	0.1	1.0
Shigellosis	109	0.9	76	0.6	1.4
Salmonellosis (NT)	1,508	12.7	842	7.1	1.8
Campylobacteriosis	1,723	14.5	555	4.7	3.1
Giardiasis	464	3.9	126	1.1	3.7
Hepatitis A	201	1.7	22	0.2	9.1

NT = non-typhoid; No. = number; SHR = surveillance to hospitalization ratio (ratio between the number of cases reported through surveillance and the number of hospitalizations observed over the same time period).

* Surveillance for *Cryptosporidium* commenced in late 1994; hospitalization data for the period 1991–1994 were censored in the calculation of the SHR for this pathogen.

† Per 100,000 persons aged ≥65 years.

Data reflect the number of cases in persons 65 years old and older reported through the state surveillance system and the number of hospitalized cases as documented by the Center for Medicare and Medicaid Services database.

### Salmonellosis

**Figure 1:**
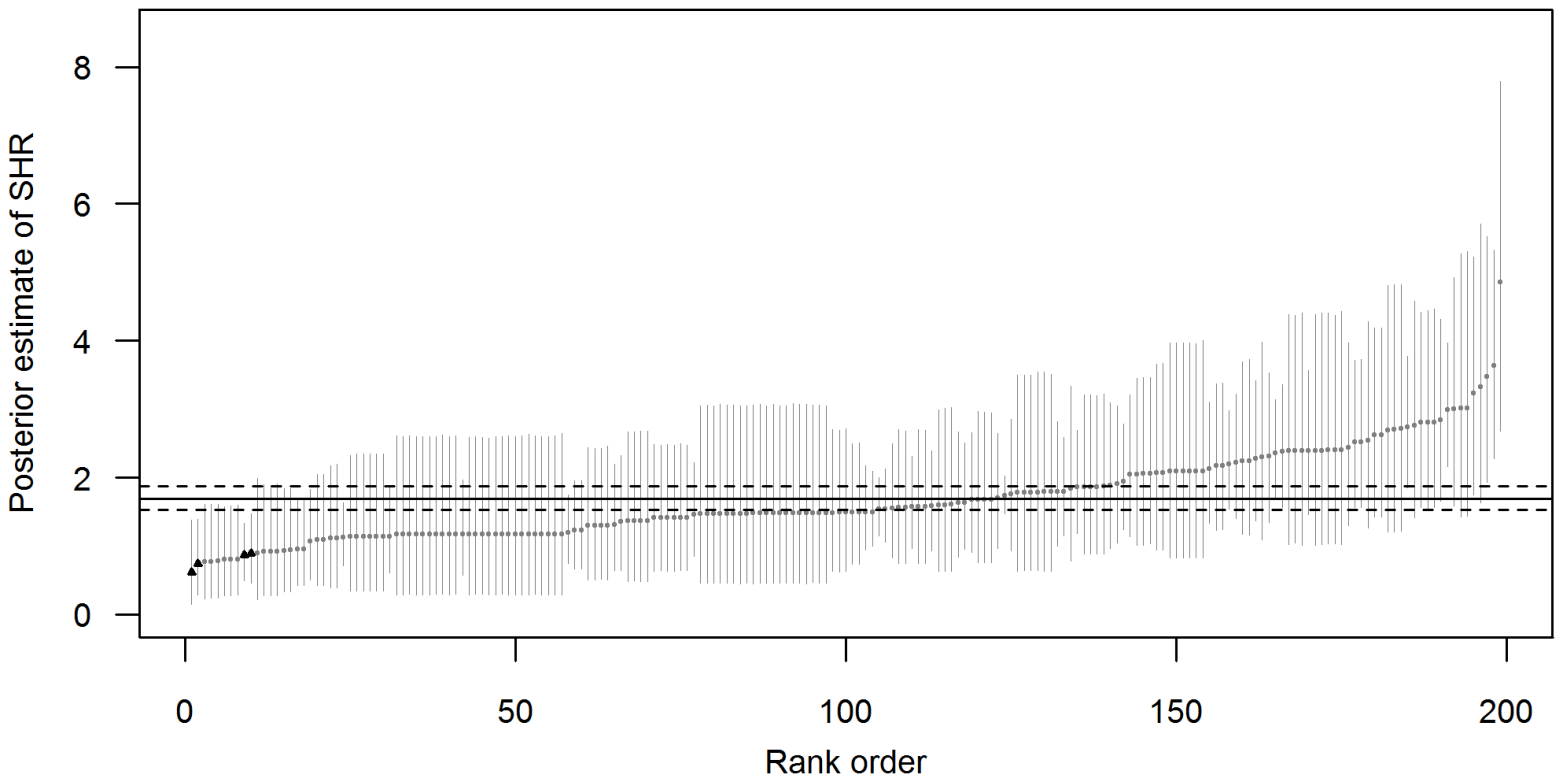
Surveillance to hospitalization ratio (SHR) for salmonellosis. For each municipality point estimates and credible intervals for the posterior estimates of the mean (So/Ho) are shown in grey. The mean estimate of the SHR for the entire state (solid line) and associated credible interval (dashed lines) are indicated as horizontal lines. Municipalities that had significantly lower SHR compared to the state mean are indicated with a black triangle.

**Figure 2:**
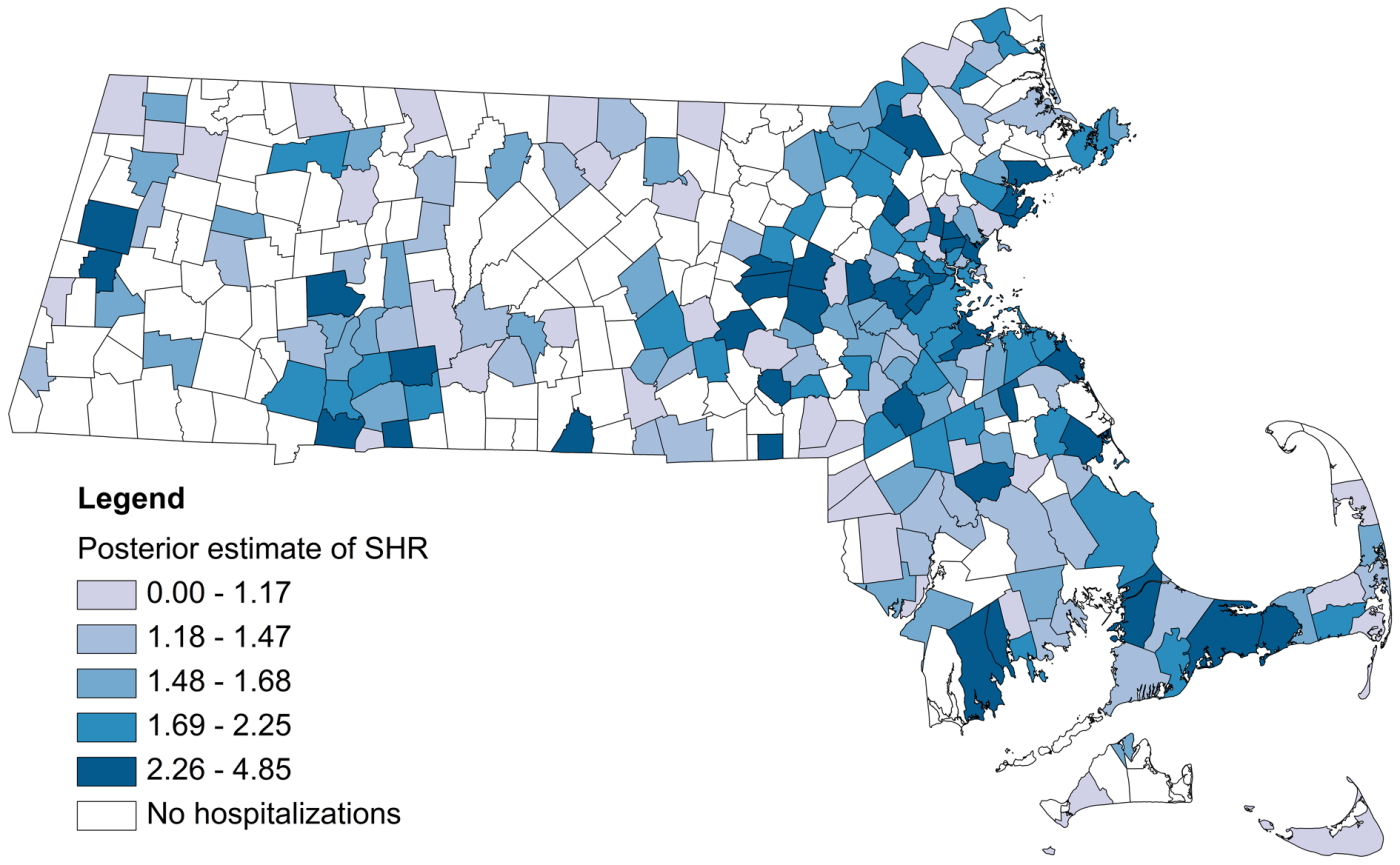
Surveillance to hospitalization ratio (SHR) for salmonellosis. Municipalities are categorized according to quintiles. For confidentiality reasons the four jurisdictions that are suspected of under-ascertaining cases have not been identified.

Between 1991 and 2004, at least one case of hospitalization due to *Salmonella* was observed in 199/351 municipalities in Massachusetts. Among 152 municipalities where there were no *Salmonella* hospitalizations, the number of cases reported to MDPH ranged from 0 to 7 (median: 0). Figures 1 and 2 show the smoothed estimates of the SHR for salmonellosis by municipality. On average, approximately 1.7 cases of salmonellosis were reported to MDPH for every 1 hospitalization with this pathogen [95% CI: 1.53, 1.87]. Four (2%) municipalities were suspected of under-reporting for this pathogen.

**Figure 3:**
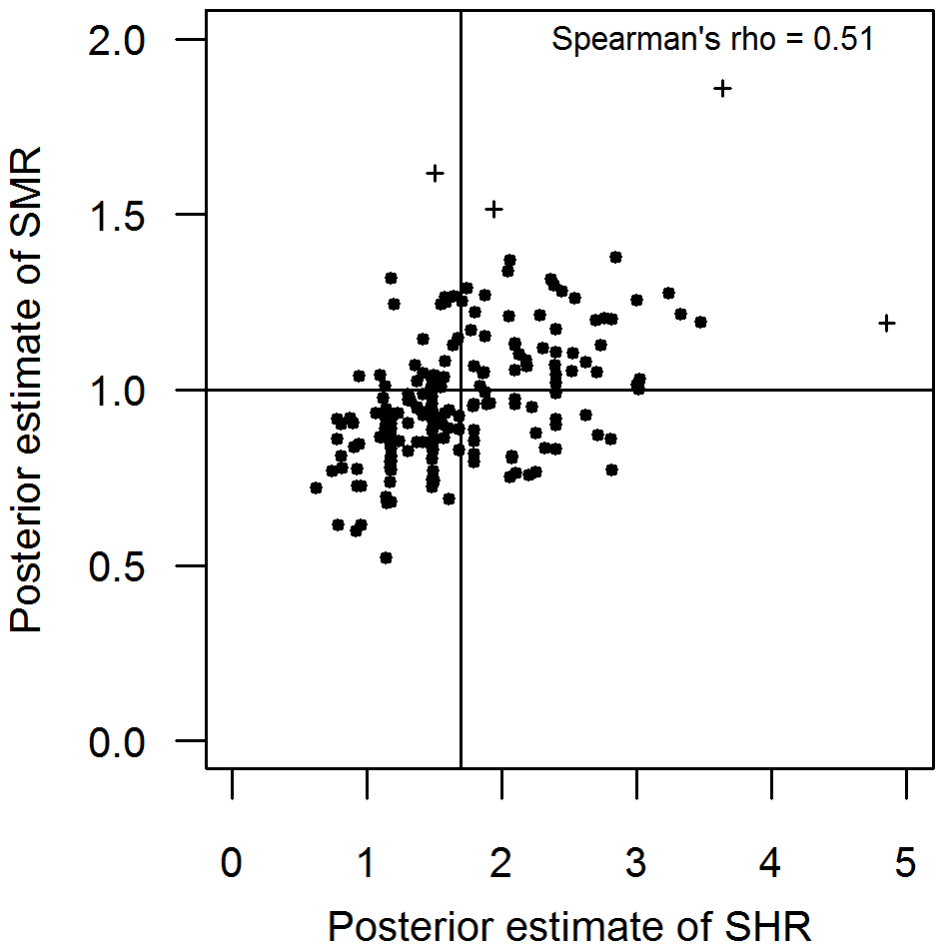
Comparison between the standardized morbidity ratio (SMR) and the surveillance to hospitalization ratio (SHR) for salmonellosis. The null hypothesis for the SMR is indicated as a horizontal line (SMR = 1). The mean estimate of the SHR for the entire state is indicated as a vertical line. Point estimates that are >3SD of the respective mean are indicated (‘+’). Exclusion of four outliers did not substantially alter the findings (rho = 0.50, p<0.001).

Figure 3 shows the relationship between the smooth estimates of the SMR and SHR for salmonellosis. There was a moderate positive relationship between the two ratios (p<0.001). Adjusting for differences in population structure, for every 10% decrease in SHR, SMR decreased by 2.74±0.23% (p<0.001, adjusted R^2^ = 0.27).

### Other infections

As shown in Table 1, approximately 9 cases of hepatitis A were reported for every hospitalization with this infection. In contrast, the number of hospitalizations with cryptosporidiosis exceeded the total number of reported cases (SHR<1), indicating overall substantial under-reporting of this particular infection.

On average, approximately 3 cases of campylobacteriosis were reported to MDPH for every case hospitalized with this infection (95% CI: 2.72, 3.55). Twenty out of 172 (11.6%) municipalities with ≥1 *Campylobacter* hospitalization were suspected of under-reporting for this pathogen. For giardiasis approximately 2 cases were reported for every case hospitalized (95% CI: 1.56, 2.50); none of the 72 municipalities with ≥1 *Giardia* hospitalization were considered suspect.

**Figure 4:**
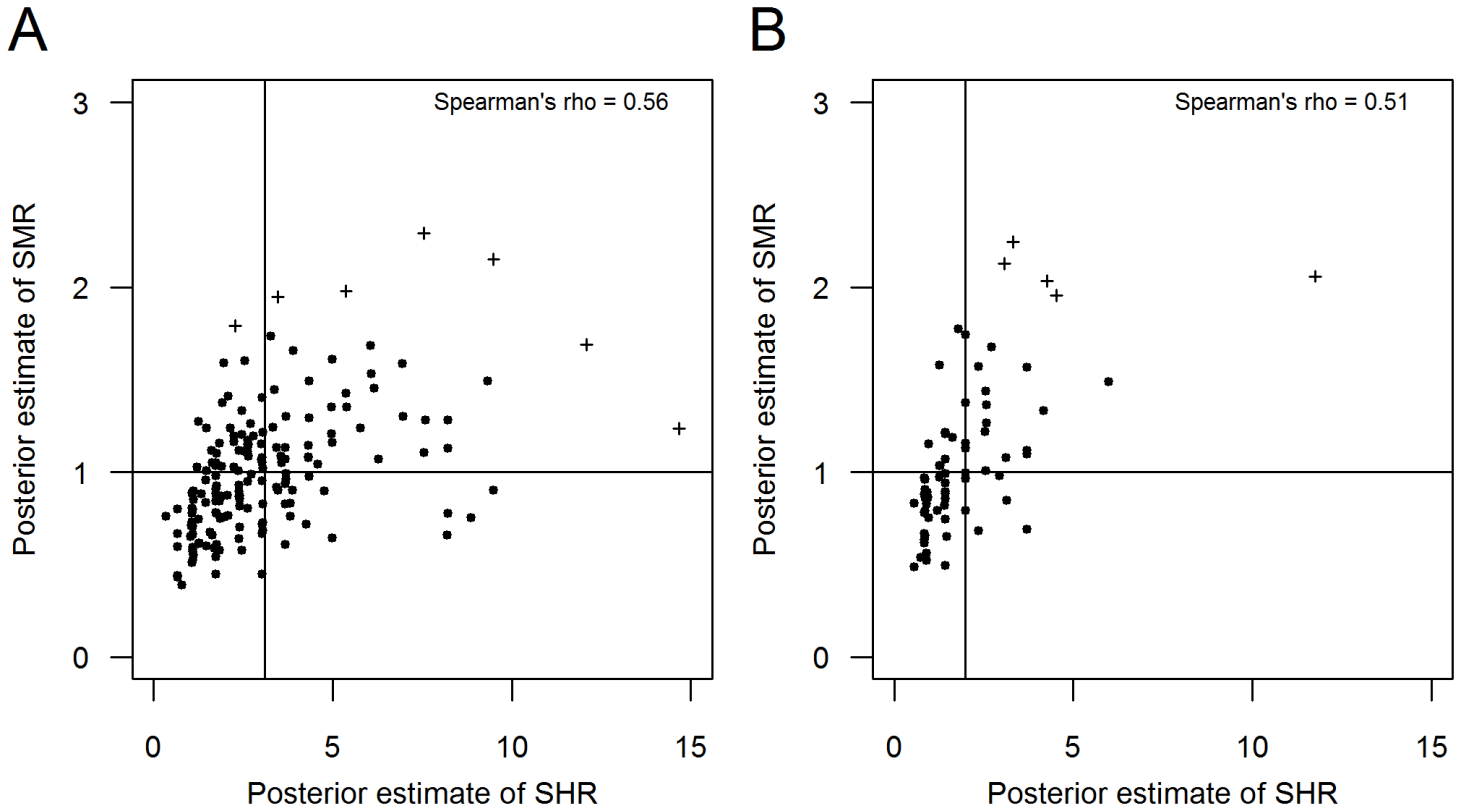
Comparison between the standardized morbidity ratio (SMR) and the surveillance to hospitalization ratio (SHR) for campylobacteriosis (A) and giardiasis (B). The null hypothesis for the SMR is indicated as a horizontal line (SMR = 1). The mean estimate of the SHR for the entire state is indicated as a vertical line for both infections. Point estimates that are >3SD of the respective mean are indicated (‘+’). Exclusion of outliers did not substantially alter the findings (*Campylobacter*: rho = 0.53, p<0.001; *Giardia*: rho = 0.60, p<0.001).

Figures 4A and 4B show the relationship between the SMR and SHR for campylobacteriosis and giardiasis, respectively. Similar to salmonellosis, we found a moderate positive association between the two ratios for both infections (p<0.001 for both infections). For every 10% decrease in SHR, SMR decreased by approximately 2.86±0.33% for *Campylobacter* (p<0.001, adjusted R^2^ = 0.33) and 4.34±0.53% for *Giardia* (βp<0.001, adjusted R^2^ = 0.48) adjusting for differences in population structure.

## Discussion

Surveillance data on food- and waterborne infections are inherently incomplete because infection typically presents as mild, self-limiting disease in the majority of those affected and requires submission of a laboratory specimen for confirmation. The extent to which surveillance systems detect such illnesses is difficult to assess in the absence of data on the “true” number of cases in the population. As a result it is difficult to ascertain whether geographic differences in reported cases represent true signals (variations in incidence) or systematic measurement error stemming from clinical (testing) and non-clinical (reporting) factors. By comparing the number of reported cases with the number of hospitalized cases for the same infection we identified municipalities that significantly deviated from the typical experience in the state and might be under-ascertaining cases. The names of these jurisdictions have not been made public for confidentiality reasons. We can reveal that jurisdictions flagged as having a low SHR were primarily older industrial cities with significant public health resource restraints that would tend to explain performance. This scenario was true for many of the municipalities in the lowest quintile of SHR (see Figure 2).

In spatial epidemiology research, standardized morbidity ratios are a common method for representing relative differences in disease risk across geographies [14]. We applied this methodology to standardize surveillance counts with respect to the elderly population, allowing comparison between each municipality and the mean rate reported for the state. Application of Markov Chain Monte Carlo simulation enabled generation of credible intervals for a plausible range of values for each municipality, while also minimizing the influence of data from small areas [14], [16]. We found marked variation in SMR for salmonellosis across the state of Massachusetts, ranging from 0.52 (low incidence relative to mean) to 1.86 (high incidence relative to mean). It is plausible that this spatial heterogeneity depicted by SMR is due to the varying risk of food-borne and waterborne disease across Massachusetts, stemming from differences in exposure levels (e.g. contact rates, pathogen dose) or distribution of population at risk (e.g. “older” municipalities). Indeed, an analysis of environmental factors associated with the rate of reported protozoan infections demonstrated links with the type of water source, income, and crowdedness, which are unevenly distributed across the state [19], [20]. We postulate however that – when applied to passive surveillance data – SMR may be confounded by inconsistent testing and reporting practices across municipalities. While testing and reporting procedures should be standardized across the state and based on comprehensive published guidelines [12], resource constraints and variations in healthcare access dictate that these guidelines are differentially applied. The extent to which such variation confounds the spatial “signal” depicted by surveillance is difficult to assess in the absence of external data against which these findings can be verified.

In this paper we introduce the surveillance to hospitalization ratio (SHR) as a complimentary technique to aid in identification of municipalities that might be under-ascertaining cases of reportable disease. Unlike the SMR, which depicts variations in “true” incidence relative to the state mean as well as (potential) artifacts created by variable testing and/or reporting practices, we suggest that differences in SHR across municipalities should principally reflect only the latter for any given infection and population (assuming hospitalization rates are consistent across municipalities). It is plausible that differences in population age-structure could contribute to spatial variation in SHR (e.g. lower SHR in municipalities with more persons aged 85 years and older). An examination of the population age-structure in municipalities with the 15 highest and 15 lowest SHRs did not support this conclusion however (see Figure S1).

We found that SHR was a significant predictor of SMR; municipalities that reported fewer cases relative to the number of hospitalizations had a lower relative risk (as estimated by SMR), adjusted for population age-structure. Importantly, this finding was consistent across all three infections studied. This observation supports our assumption that the spatial “signal” depicted by surveillance is in part influenced by inconsistent testing and reporting practices across the state and that SHR may be helpful in discerning which jurisdictions may be underperforming in terms of identifying cases of reportable disease.

We found that SHR varied considerably by disease, consistent with our hypothesis that this ratio also reflects pathogen specific processes (e.g. differences in virulence). For instance, the crude SHR varied from 0.5 for cryptosporidiosis (more hospitalized than reported) to 9.1 for hepatitis A infection (more reported than hospitalized). The low SHR for cryptosporidiosis clearly indicates under-reporting or inability to follow up positive laboratory results for confirmation of the case, since – per the Massachusetts guidelines [12] – all of the hospitalized cases should have been reported to the BOH. The SHR of 9.1 for hepatitis A likely reflects both the limited morbidity associated with infection, and possibly the recognized problem of false positive laboratory results arising from the use of “hepatitis panels” in elderly patients with abnormal liver function tests [21].

Few studies report simultaneous data for surveillance and hospitalization cases against which the crude SHRs calculated here can be compared. Inference from data presented in two studies undertaken in the general population in Denmark [22] and in states participating in FOODNET in the United States [23] yield estimates of 3.6 and 5.6 for salmonellosis, respectively. Likewise, these studies suggest that the SHR for campylobacteriosis is 5.6 and 6.9. These studies agree with our finding of a lower SHR for *Salmonella* compared to *Campylobacter* (1.8 vs 3.1 in Massachusetts), albeit with higher overall values reported in these other studies. The reason for the latter may relate to the fact that our study focused on an elderly population that is more likely to be hospitalized following infection. This hypothesis is supported by a California study from which we can infer that the SHR for salmonellosis among persons aged <5, 5–64 and ≥65 years was 4.2, 3.6 and 1.2, respectively [24]. Indeed, even within the ≥65 years age category we found that SHR declined steeply with increasing age (see Table S1). In sum, our findings suggest that pathogen- and population-specific (age-standardized) assessment of SHR is warranted and requires further research.

### Limitations

There are several limitations to this study. The use of hospital discharge data to evaluate public health surveillance systems is not a new proposition; researchers have demonstrated the utility of such data when evaluating surveillance for infectious [10], [25], non-infectious [26], environmental health outcomes [27]. Nevertheless, the accuracy and completeness of data contained in Medicare, hospital discharge and other administrative databases has been called into question in respect to various infectious diseases [28]–[30]. The extent to which these concerns hold true for gastrointestinal infections is not known. It is possible that diagnoses made on the basis of laboratory results obtained after admission are not coded in the discharge record. We included all 10 diagnostic codes in our study since – per the Massachusetts guidelines [12] – notifiable pathogens should be reported whenever they are detected, even if they are not the primary reason for illness. A majority of Medicare records used in this study contained the diagnostic codes of interest in position 1 or 2 (see Table S2). Further research is needed to characterize the accuracy and completeness of diagnostic coding for gastrointestinal infections. In this study, analysis was limited to the period 1991–2004, reflecting availability of data under data sharing agreements between Tufts University and the owners of the data. It is unlikely that the findings would be invalidated by inclusion of more recent data.

Our study focused on persons aged 65 years and older, owing to the fact that Medicare records include information on ∼96% of the US elderly population [31]. They therefore provide excellent coverage of the population under surveillance in this age range. Unfortunately, Medicare databases do not permit application of these methods to a younger cohort since enrollment of such persons in the program is based on having a disability. While the rate of testing and diagnosis were biased by the greater likelihood of being sicker, hospitalized and having a test done in the elderly population, we maintain that within an age strata, the relationship between surveillance counts and the number of people hospitalized should remain fairly consistent for each pathogen.

It is likely that data from the elderly do not reflect the overall performance of a surveillance system. The spectrum of environmental exposures in elderly differs from the general population and – given the severity of infection – they might have a greater likelihood of being diagnosed especially when ill enough to be hospitalized. Several experts have called for targeted monitoring of gastrointestinal illness in this particular sub-population given that they are especially vulnerable to severe outcomes of gastrointestinal illness [32]–[34]. Additionally, published guidelines support routine stool cultures on all patients admitted to the hospital with community-acquired diarrhea and all 65 years of age or older with pre-existing conditions who develop diarrhea 72 or more hours after admission [35]. It therefore seems reasonable to undertake measures to strengthen disease surveillance activities for this specific population.

While it is conventional to consider an SMR = 1 as the null value (obtained when observed and expected values are the same), defining appropriate cut-points for SHR was more challenging because the relationship between the number of hospitalized and reported cases was unknown at the outset. We defined a municipality as potentially under-reporting if the upper limit of the 95% credible interval for the municipality did not overlap with the lower limit for the entire state. This is a conservative approach and may have resulted in misclassification. With further research it should be possible to define appropriate “cut-off” values for SHR, facilitating improved detection of municipalities that deviate substantially from the typical experience. In any case state health departments may choose to raise or lower such threshold depending on their needs and resources (e.g. investigate municipalities in lowest quintile).

### Future directions

We propose to further explore the usability of routinely collected health data, such as surveillance and hospitalization records in an integrative manner. Proven successful, these integrated metrics can be employed periodically using temporally aggregated data and eventually adapted to monitor performance of surveillance systems in “real time”. Such a metric would be similar to the Standardized Infection Ratio, which is used in infection control epidemiology and aids in benchmarking incidence of hospital-acquired infection (HAI) in single hospitals against national average rates of HAI [36]–[38]. We suggest that such an integrated approach to gastroenteritis surveillance might have a potential to assess the impact of changes in clinical algorithms (such as expansion of managed care) and testing methods on disease surveillance, effects of population migration patterns on health care utilization [39], and state-wide intervention strategies or nation-wide policy change impacts [40].

## Conclusions

The Centers for Disease Control and Prevention (CDC) outlines a framework for evaluating public health surveillance activities, a stated purpose of which is to develop recommendations for improving data quality [41]. The expansion of the use of electronic health records promises more direct and complete reporting of notifiable diseases with ability to supplement and evaluate traditional surveillance systems [9], [10], [42]. Incorporating assessment of SHR during periodic evaluations of surveillance activities could provide an objective and practical means to identify jurisdictions that may be under-ascertaining cases of reportable disease. Benchmarking performance of municipalities to the rest of the state could help guide attention to jurisdictions that may need assessment for resources and priority setting.

## Supporting Information

Figure S1 **Surveillance to hospitalization ratio (SHR) for salmonellosis, by age-category.** Bars represent the 15 lowest and 15 highest ranking municipalities (corresponding to Figure 2). Within the ≥65 age category, the proportion aged 65–74 years (dark grey), 75–84 years (medium grey) and 85 years and older (light grey) are shown.
(TIF)

Table S1 **Salmonellosis in Massachusetts, by age category, 1991–2004.** Data reflect the number of cases in persons 65 years old and older reported through the state surveillance system and the number of hospitalized cases as documented by the Center for Medicare and Medicaid Services database.
(DOCX)

Table S2 **Salmonellosis hospitalizations in Massachusetts, by diagnostic code position, 1991–2004.** Data reflect the first occurrence of a non-typhoid *Salmonella* ICD-9-CM code (003.X) in 1 of 10 code positions in persons 65 years old and older, as documented by the Center for Medicare and Medicaid Services database.
(DOCX)
