# Correction: Hospitalization Records as a Tool for Evaluating Performance of Food- and Water-Borne Disease Surveillance Systems: A Massachusetts Case Study

**DOI:** 10.1371/journal.pone.0104143

**Published:** 2014-07-28

**Authors:** The PLOS ONE Staff

There is an error in the author affiliations. Please refer to the correct author affiliations below:

Siobhan M. Mor

Farm Animal and Veterinary Public Health, Faculty of Veterinary Science, The University of Sydney, Sydney, New South Wales, Australia

Siobhan M. Mor

Marie Bashir Institute for Infectious Diseases and Biosecurity/School of Public Health, The University of Sydney, Sydney, New South Wales, Australia

Siobhan M. Mor, Elena N. Naumova

Department of Public Health and Community Medicine, School of Medicine, Tufts University, Boston, Massachusetts, United States of America

Elena N. Naumova

Department of Civil and Environmental Engineering, School of Engineering, Tufts University, Medford, Massachusetts, United States of America

Alfred DeMaria Jr.

Bureau of Infectious Diseases, Massachusetts Department of Public Health, Boston, Massachusetts, United States of America
