# Supplementary figures and images for: Hospitalization Records as a Tool for Evaluating Performance of Food- and Water-Borne Disease Surveillance Systems: A Massachusetts Case Study
Source: PLoS One. 2014 Apr 16;9(4):e93744. doi: 10.1371/journal.pone.0093744 (PMC3989214; doi:10.1371/journal.pone.0093744)

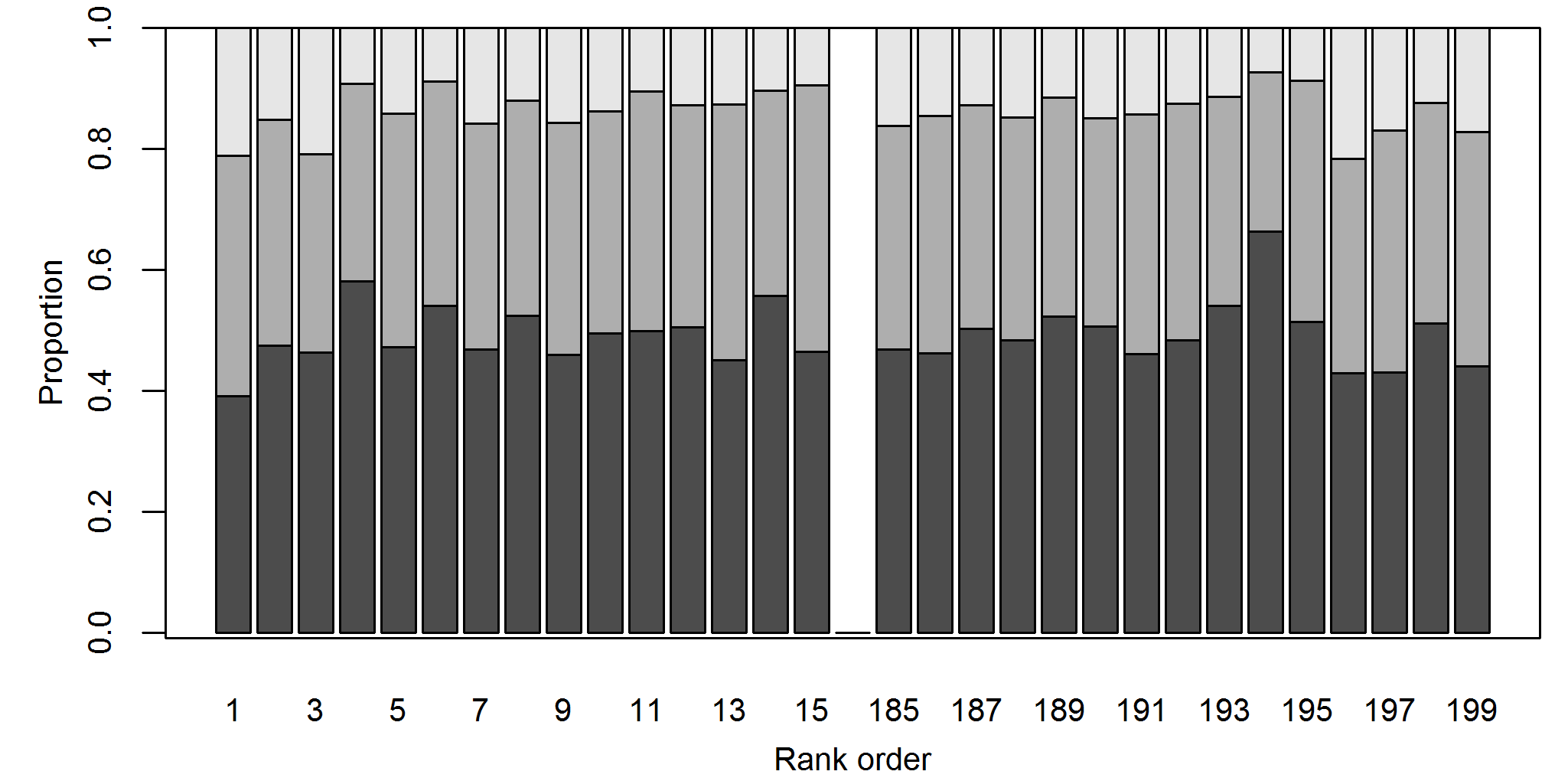

Supplement: Figure S1 — Surveillance to hospitalization ratio (SHR) for salmonellosis, by age-category. Bars represent the 15 lowest and 15 highest ranking municipalities (corresponding to Figure 2). Within the ≥65 age category, the proportion aged 65–74 years (dark grey), 75–84 years (medium grey) and 85 years and older (light grey) are shown. (TIF) [file pone.0093744.s001.tif]
