# Supplementary material for: Hospitalization Records as a Tool for Evaluating Performance of Food- and Water-Borne Disease Surveillance Systems: A Massachusetts Case Study
Source: PLoS One. 2014 Apr 16;9(4):e93744. doi: 10.1371/journal.pone.0093744 (PMC3989214; doi:10.1371/journal.pone.0093744)
Supplement: Table S1 — Salmonellosis in Massachusetts, by age category, 1991–2004. Data reflect the number of cases in persons 65 years old and older reported through the state surveillance system and the number of hospitalized cases as documented by the Center for Medicare and Medicaid Services database. (DOCX) [file pone.0093744.s002.docx]

| Age category | Surveillance | | Hospitalization | | SHR |
| --- | --- | --- | --- | --- | --- |
|  | No. of cases | Annual incidence† | No. of cases | Annual incidence† |  |
| 65-69 | 376 | 11.9 | 139 | 4.4 | 2.7 |
| 70-74 | 353 | 12.0 | 164 | 5.6 | 2.2 |
| 75-79 | 320 | 12. 8 | 175 | 7.0 | 1.8 |
| 80-84 | 257 | 14.7 | 187 | 10.7 | 1.4 |
| 85+ | 202 | 13.1 | 177 | 11.4 | 1.1 |
| No. = number; SHR = surveillance to hospitalization ratio (ratio between the number of cases reported through surveillance and the number of hospitalizations observed over the same time period)  † Per 100,000 persons | | | | | |
