# Supplementary material for: Hospitalization Records as a Tool for Evaluating Performance of Food- and Water-Borne Disease Surveillance Systems: A Massachusetts Case Study
Source: PLoS One. 2014 Apr 16;9(4):e93744. doi: 10.1371/journal.pone.0093744 (PMC3989214; doi:10.1371/journal.pone.0093744)
Supplement: Table S2 — Salmonellosis hospitalizations in Massachusetts, by diagnostic code position, 1991–2004. Data reflect the first occurrence of a non-typhoid Salmonella ICD-9-CM code (003.X) in 1 of 10 code positions in persons 65 years old and older, as documented by the Center for Medicare and Medicaid Services database. (DOCX) [file pone.0093744.s003.docx]

| Diagnostic  code position | No. of cases | Proportion |
| --- | --- | --- |
| 1 | 486 | 0.58 |
| 2 | 105 | 0.12 |
| 3 | 62 | 0.07 |
| 4 | 61 | 0.07 |
| 5 | 59 | 0.07 |
| 6 | 20 | 0.02 |
| 7 | 22 | 0.03 |
| 8 | 18 | 0.02 |
| 9 | 9 | 0.01 |
| 10 | 0 | 0 |
| Total | 842 | 1.0 |
| No. = number | | |

**Supplementary figure (legend)**

**Figure S1. Surveillance to hospitalization ratio (SHR) for salmonellosis, by age-category.** Bars represent the 15 lowest and 15 highest ranking municipalities (corresponding to Figure 2). Within the ≥65 age category, the proportion aged 65-74 years (dark grey), 75-84 years (medium grey) and 85 years and older (light grey) are shown.
